# Supplementary material for: Peak Tibiofemoral Contact Forces Estimated Using IMU-Based Approaches Are Not Significantly Different from Motion Capture-Based Estimations in Patients with Knee Osteoarthritis
Source: Sensors (Basel). 2023 May 4;23(9):4484. doi: 10.3390/s23094484 (PMC10181595; doi:10.3390/s23094484)
Supplement: Supplementary file 1 [file sensors-23-04484-s001.zip › sensors-2349504-supplementary.pdf]

Supplementary Materials:

PPCA estimated joint moments (Figure S1): The customized PPCA KOA-based model showed an average RMSE of the estimated hip flexion, adduction and rotation moment of 0.15 ± 0.04 Nm/kg, 0.16 ± 0.04 Nm/kg and 0.03 ± 0.06 Nm/kg, respectively with R^2 of 0.77 ± 0.20, 0.79 ± 0.15 and 0.59 ± 0.24, respectively. And the average RMSE of the estimated knee flexion and ankle flexion moment of 0.12 ± 0.06 Nm/kg and 0.15 ± 0.04 Nm/kg, respectively with R^2 of 0.69 ± 0.15 and 0.92 ± 0.35, respectively.

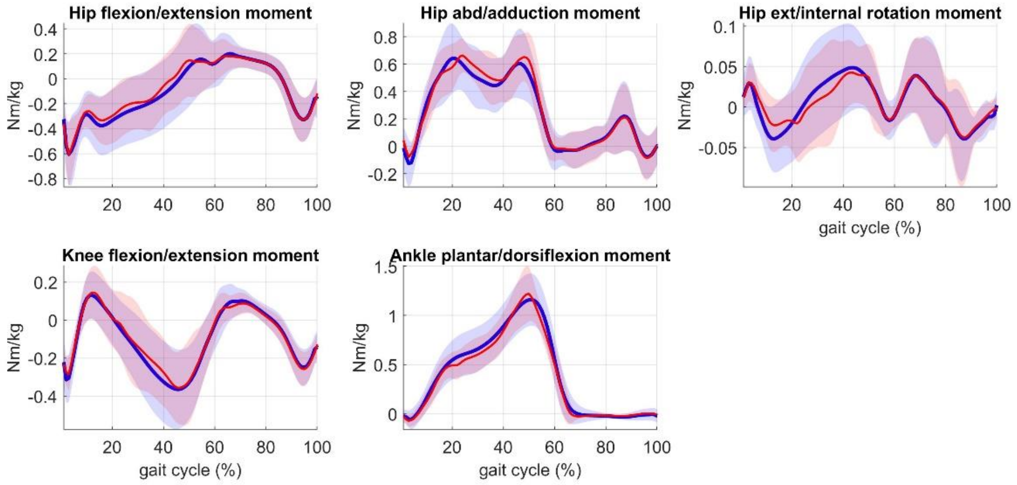

Figure S1. MoCap (blue) vs estimated (red) joint moments of the PPCA KOA population-based model.

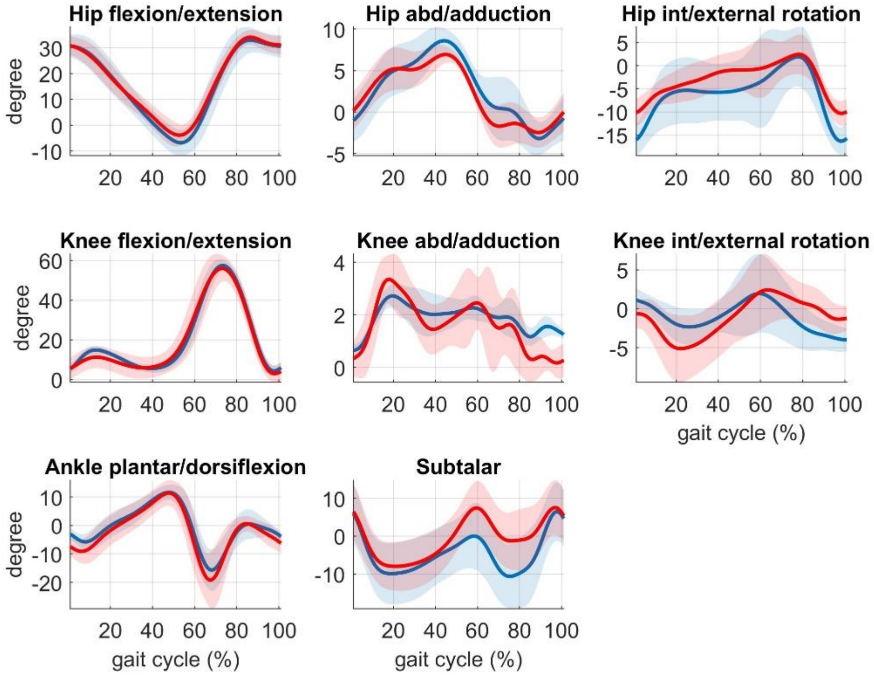

Figure S2. MoCap (blue) vs InCap (red) joint kinematics comparison

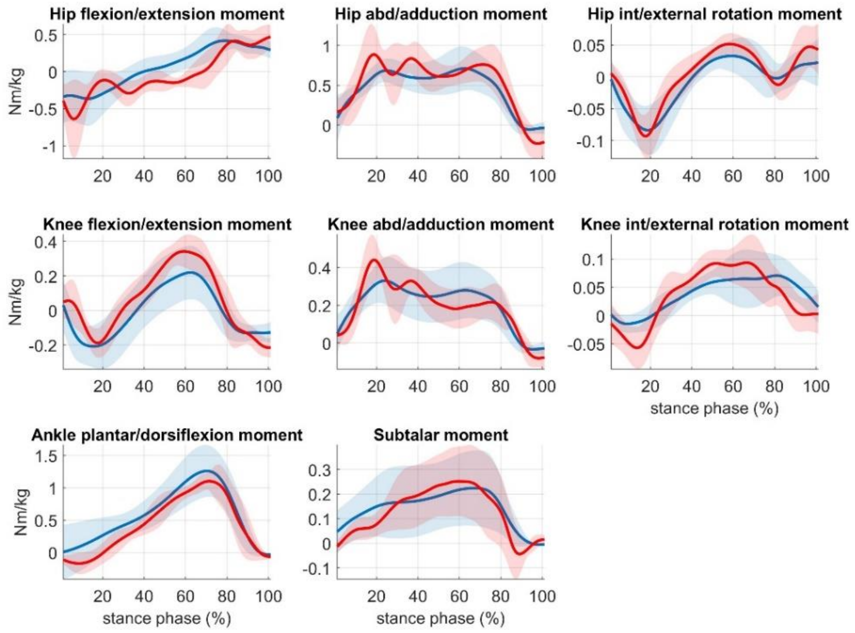

Figure S3. MoCap (blue) vs InCap (red) joint moments comparison

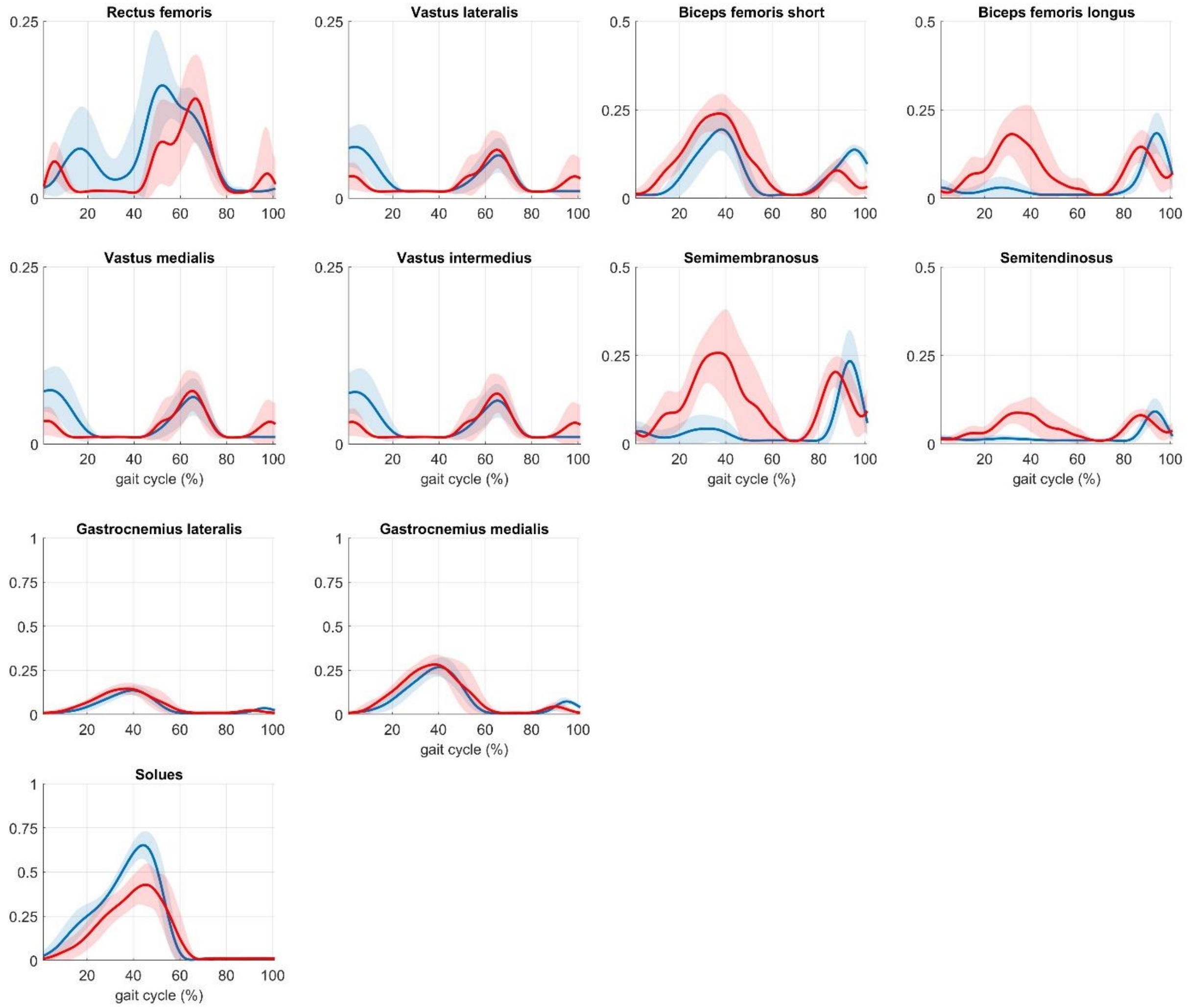

Figure S4. MoCap (blue) vs InCap (red) muscle activation comparison (main muscles).

Table S1. Root mean square error (RMSE) and coefficient of determination (R2) of joint kinematics, joint momentsa and muscle activation between MoCap and InCap.

**Joint kinematics**	**RMSE (°)**		**R2**	
	**Mean**	**Std**	**Mean**	**Std**
Hip flexion/extension	5.04	1.70	0.95	0.05
Hip abd/adduction	1.88	0.56	0.86	0.10
Hip int/external rotation	4.73	2.50	0.83	0.21
Knee flexion/extension	4.78	1.52	0.92	0.09
Knee abd/adduction	1.42	0.31	0.45	0.17
Knee int/external rotation	4.44	1.86	0.47	0.20
Ankle plantar/dorsiflexion	5.12	1.25	0.84	0.17
Subtalar	4.80	1.36	0.70	0.13

**Joint moments**	**RMSE (Nm/kg)**		**R2**	
	**Mean**	**Std**	**Mean**	**Std**
Hip flexion/extension	0.31	0.20	0.57	0.12
Hip abd/adduction	0.31	0.14	0.69	0.20
Hip int/external rotation	0.03	0.01	0.73	0.12
Knee flexion/extension	0.14	0.07	0.74	0.19
Knee abd/adduction	0.14	0.04	0.69	0.18
Knee int/external rotation	0.04	0.02	0.65	0.26
Ankle plantar/dorsiflexion	0.32	0.19	0.75	0.17
Subtalar	0.16	0.08	0.52	0.31

**Muscle activation**	**RMSE (0-1)**		**R2**	
	**Mean**	**Std**	**Mean**	**Std**
Gastrocnemius lateralis	0.033	0.020	0.81	0.18
Gastrocnemius medialis	0.065	0.038	0.80	0.20
Soleus	0.130	0.041	0.73	0.18
Biceps femoris short	0.076	0.039	0.48	0.30
Biceps femoris long	0.095	0.037	0.12	0.14
Semimembranosus	0.141	0.054	0.12	0.11
Semitendinosus	0.048	0.016	0.10	0.12
Vastus lateralis	0.026	0.015	0.44	0.34
Vastus medialis	0.024	0.014	0.45	0.34
Vastus intermedius	0.025	0.014	0.44	0.34
Rectus femoris	0.064	0.029	0.33	0.25

Table S2. p and q-value for testing significancy (< 0.05) of differences in Knee contact forces peak and impulse—significant different values in bold.

		MoCap vs InCap	
		**p-value**	**q-value**
Peak 1	**Total**	0.4623	1.6816
	**Medial**	0.1221	0.6218
	**Lateral**	0.1868	0.7925
Peak 2	**Total**	0.0143	0.1212
	**Medial**	0.0316	0.2009
	**Lateral**	0.5463	1.7388
Impulse	**Total**	0.0020	**0.0252**
	**Medial**	0.0010	**0.0252**
	**Lateral**	0.8799	2.4894
